# Data in support of preparation and functionalization of a clickable polycarbonate monolith

**DOI:** 10.1016/j.dib.2016.02.020

**Published:** 2016-02-16

**Authors:** Yuanrong Xin, Junji Sakamoto, André J. van der Vlies, Urara Hasegawa, Hiroshi Uyama

**Affiliations:** a Department of Applied Chemistry, Graduate School of Engineering, Osaka University, Suita 565-0871, Japan; b Frontier Research Center, Graduate School of Engineering, Osaka University, Suita 565-0871, Japan; c Frontier Research Base for Young Researchers, Graduate School of Engineering, Osaka University, Suita 565-0871, Japan

## Abstract

This data article provides supplementary figures to the research article entitled, “Phase separation approach to a reactive polycarbonate monolith for “click” modifications” (Xin et al., Polymer, 2015, doi:10.1016/j.polymer.2015.04.008). Here, the nitrogen adsorption/desorption isotherms of the prepared porous polycarbonate monolith are shown to classify its inner structure and calculate the specific surface area. The monoliths were modified by using the thiol-ene click chemistry and the olefin metathesis, which was examined by contact angle measurements, FT-IR, solid state ^13^C NMR spectroscopy as well as thermogravimetric analysis.

## 1 **Specifications Table**

**Table:** 

Subject area	*Materials science*
More specific subject area	*Polymeric porous material*
Type of data	*Figure, image (contact angle measurement), spectra*
How data was acquired	*Nitrogen adsorption/desorption, contact angle measurement, solid state*^*13*^*C NMR, FT-IR, TGA*
Data format	*Raw*
Experimental factors	*Polymeric monoliths need to be smashed into powder before solid state*^*13*^*C NMR and TGA measurements.*
Experimental features	*Characterization of the prepared polymeric monolith and its structural change after functionalization reactions*
Data source location	*Osaka University, Osaka, Japan*
Data accessibility	*The data is available with this article and is related to*[1].

## 2 **Value of the data**

• The data presented here are useful as the references and comparisons for other research groups who are working at the fabrication, characterization and surface-structure design of porous materials.
• The nitrogen adsorption/desorption isotherms can be employed to confirm the inner structure and calculate the specific area of porous materials.
• Data of contact angle measurements can be used to examine the surface polarity change of the modified monolithic materials after introducing polar functional groups.
• Data of FT-IR spectra, solid state ^13^C NMR spectra as well as thermogravimetric analysis (TGA) are significant to characterize monolithic materials with chemical structure change and gain insights about the effect caused by the olefin metathesis.

## 3 Data

### 3.1 Nitrogen adsorption/desorption isotherms of polycarbonate monolith

**Fig. 1:**
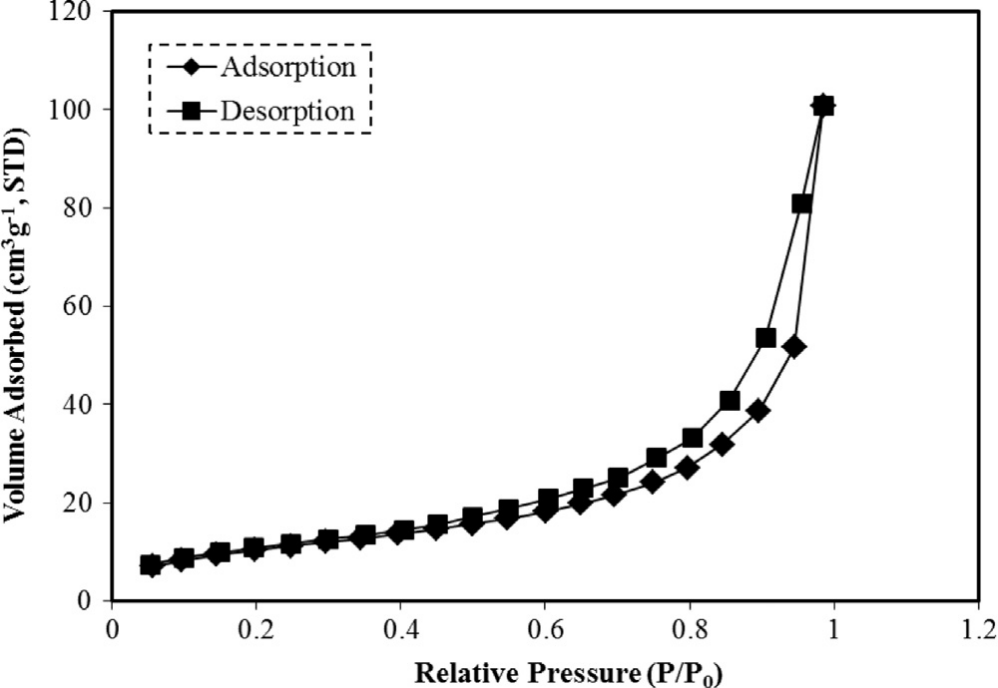
Nitrogen adsorption/desorption isotherms of BM-PC monolith.

Polycarbonate monolith with allyl groups (BM-PC) was prepared through phase separation method as described in Ref. [1]. The resultant monolith possesses unique continuous interconnected porous structure. Fig. 1 shows the nitrogen adsorption/desorption isotherms of the BM-PC monolith measured with a NOVA 4200e Surface Area & Pore Size Analyzer (Quantachrome Instruments) at 77 K. Before the measurements, the sample was degassed at 100 °C under vacuum for at least 6 h. According to the IUPAC classification, this adsorption curve is of type II with the H3 type hysteresis loop in the *P*/*P*_0_ range from 0.4 to 1.0, which is characteristic of macroporous absorbents. The specific surface area of the monolith was calculated to be 145 m^2^/g by using the Brunauer Emmett Teller (BET) equation.

### 3.2 Contact angle measurement of BM-PC monolith

**Fig. 2:**
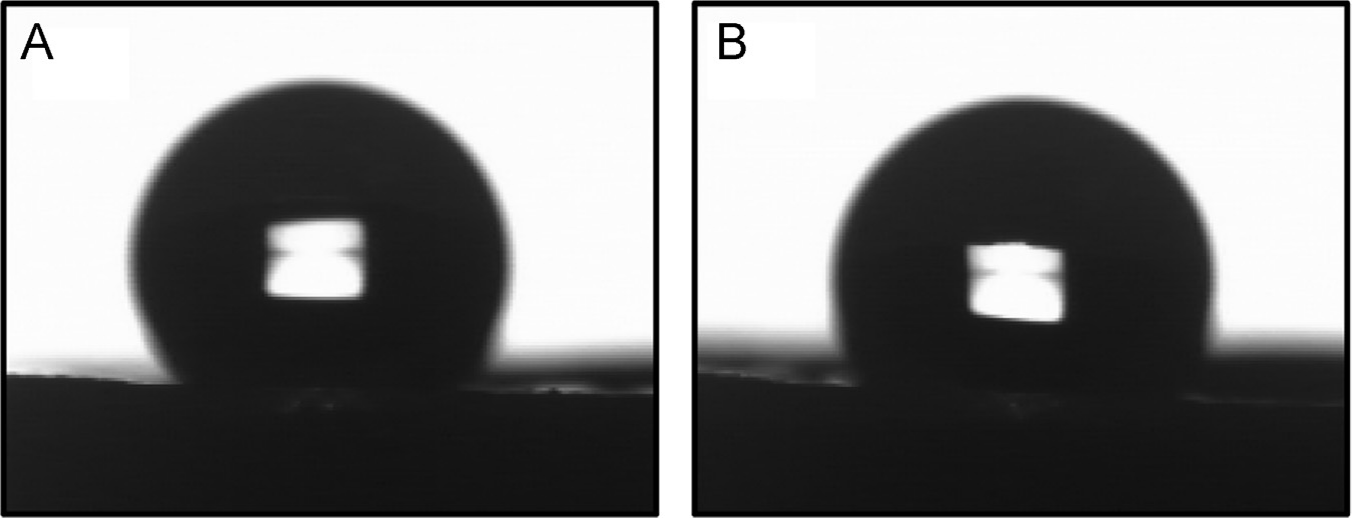
Water droplets on BM-PC monolith before (A) and after the thiol-ene click reaction (B).

The surface polarity of BM-PC monolith increases after the thiol-ene reaction due to the introduction of hydroxyl groups. As shown in Fig. 2, the contact angle measurements were carried out by sessile drop method with a Drop Master DM300 instrument (Kyowa Interface Science Co., Ltd.) using FAMAS Basic software. Specifically, 1 µL of water drop was deposited on the flat surface of polycarbonate monolith before and after the thiol-ene click reaction. The contact angle decreased from 123.3° to 112.7° as a result of the increased polarity caused by the thiol-ene reaction. Each was measured three times to confirm the reproducibility.

### 3.3 FT-IR spectra of BM-PC monolith

**Fig. 3:**
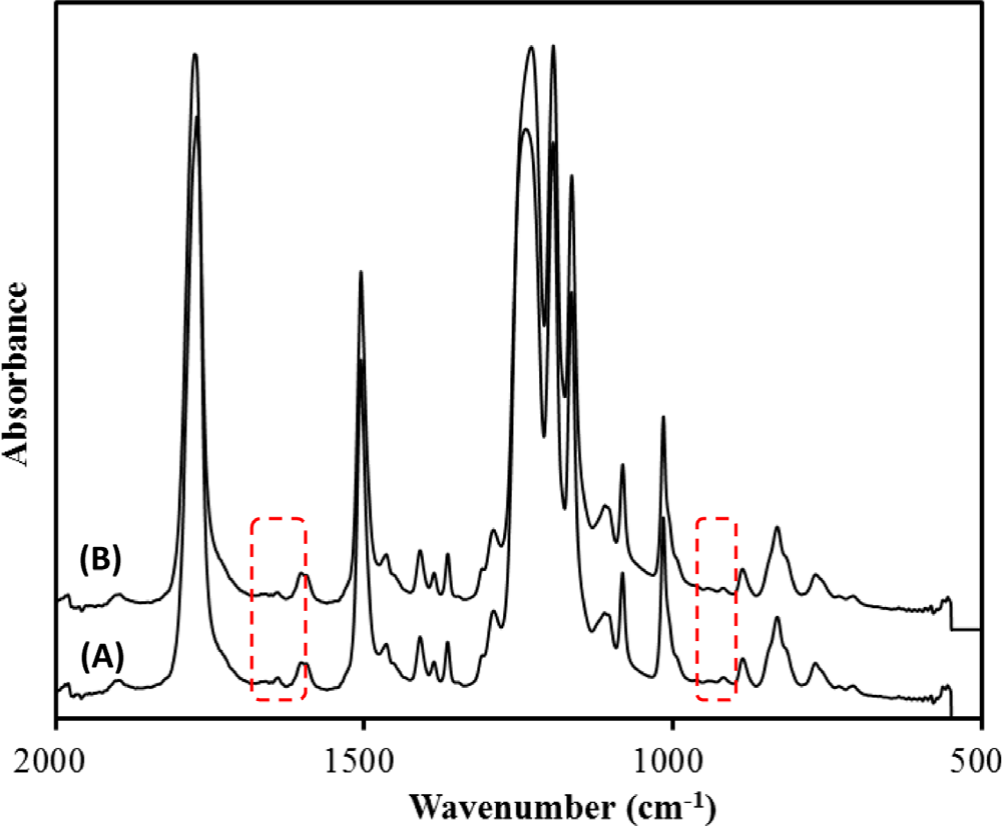
FT-IR spectra of BM-PC monolith before (A) and after the olefin metathesis (B). The regions where C=C vibrational signals typically appear are highlighted.

In attempt to prove the structural change of BM-PC monolith caused by the olefin metathesis, FT-IR spectra were measured by the attenuated total reflectance (ATR) method using Thermo Scientific Nicolet iS5 with iD5 ATR accessory (Fig. 3). However, no significant change was observed in the spectra due to the detection limit of the present measurements. This indicates a low degree of crosslinking as expected from the low density of the allyl groups on the monolith.

### 3.4 Solid state ^13^C NMR spectra of BM-PC monolith

**Fig. 4:**
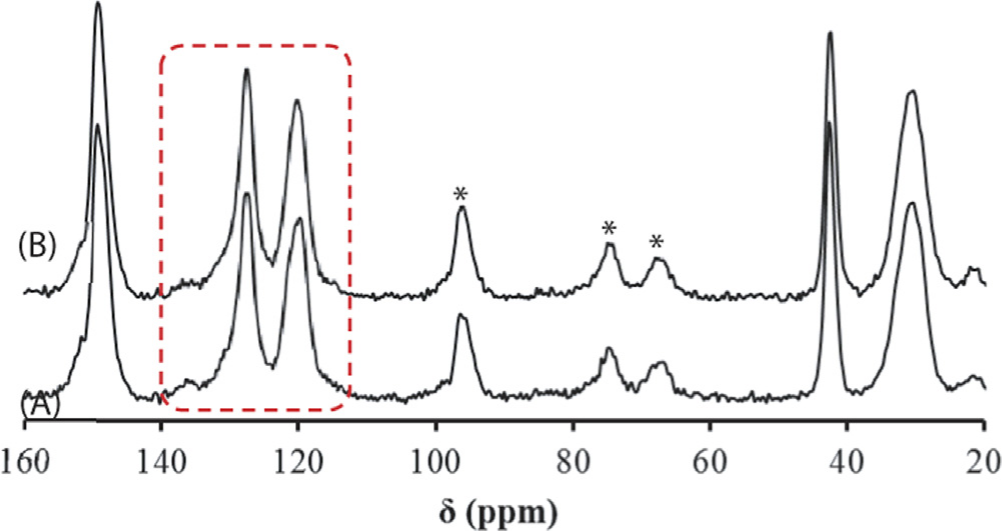
Solid-state ^13^C-NMR spectra of BM-PC monolith before (A) and after the olefin metathesis (B). Signals associated with the olefinic carbons are supposed to appear in the highlighted region. Spinning side bands are starred.

Solid state ^13^C NMR spectra of BM-PC monoliths before and after the olefin metathesis were performed using Chemagnetics 300 MHz CMX 300 Spectrometer (Bruker Avance III 600WB). The monoliths were smashed into powder prior to the measurements. As shown in Fig. 4, the internal crosslinking of BM-PC monolith caused little change in the spectra due to the detection limit here as well. This result supports the low degree of crosslinking indicated by the FT-IR measurements.

### 3.5 Thermogravimetric analysis (TGA) of BM-PC monolith

**Fig. 5:**
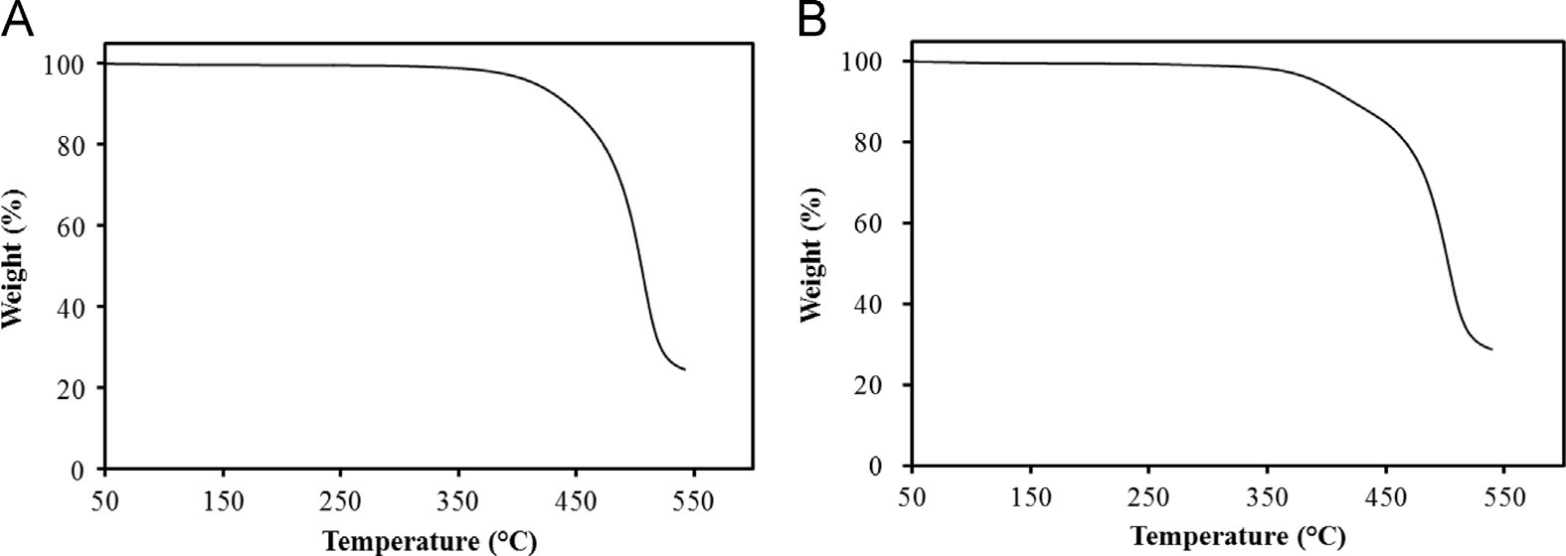
TGA curves of BM-PC monolith before (A) and after the olefin metathesis (B).

In Fig. 5, TGA was performed using BM-PC monoliths before and after the olefin metathesis with an EXSTAR TG/DTA 7200 thermogravimetric analyzer. The monoliths were smashed into powder beforehand and heated from 40 to 550 °C at a heating rate of 10 °C/min, under a steady flow of nitrogen of 300 mL/min. The crosslinking caused a significant difference in the degradation profile and the total weight loss was found to be 75.5% and 71.2% before and after the olefin metathesis, respectively.
